# Progress in Surgery

**Published:** 1903-06-06

**Authors:** 


### Progress in Surgery.

### HERNIA.

The operative treatment of hernia in infancy is not generally considered with favour by surgeons. The tendency of course in the great majority of cases is for spontaneous cure to take place after the use of a truss. There are many cases in which the truss is very irksome, the fitting difficult, and the patient and intelligent application and care necessary on the part of parents cannot be relied upon, more especially is this the case among the poorer classes, and in these cases the early treatment by operation has much to recommend it. Kennedy[1] records 60 cases in which he has operated under 5 years of age, many of these being under 12 months. Children bear the operation well, lengthy and elaborate methods are unnecessary, as at that age the natural tendency is to re-establishment of the normal condition of the canal once the sac has been removed and the contents can no longer escape. The increasingly satisfactory results of hernia operations in adults have made surgeons more inclined to extend the benefits so obtained to the extremes of life, at which periods trusses are more troublesome to wear and more difficult to render effectual than in early and middle adult life. Trusses in infants are often, and in the old still more often, a source of much trouble. There are many old men with large troublesome and irreducible herniæ which by operation, if not permanently cured, could at least be made amenable to a truss. In many such cases the great deterrent to operation is the fear of the anæsthetic. If these cases could be satisfactorily dealt with under local anæsthesia many more would be inclined to undergo operation. Bodine[2] advocates local anæsthesia, and cites 48 cases in which he has operated with cocain. He never injects more than half a grain, and uses a ·25 per cent. solution which allows of considerable fluid infiltration without the danger of toxic effects. He points out that all the parts concerned in the operation are supplied by three nerves, the ilio-hypogastric, the ilio-inguinal, and the genital branch of the genito-crural; he injects the sheath of these whereby he finds that practically all the structures handled are rendered anæsthetic. In operating under cocain vessels should not be divided if they can be avoided, as the division and ligature causes sharp pain, and if pain be inflicted at the commencement of the operation the patient may become demoralised. Large amounts of omentum may be ligatured and removed without causing pain if no traction be exerted on it. The gut may be freely handled without pain; in one case he removed the appendix without causing any pain. Dry pads inserted into the abdomen cause pain by irritating the parietal peritoneum. Moist sponges should be used if necessary to keep back the intestines. In three cases of double hernia the second side was operated on with local anæsthesia at the patients' request.

J. H. Nicholl[3] advocates a new method of dealing with femoral hernia which he has devised, the chief feature of which is his mode of closing the ring; he drills two holes through the pubic bone, through each of these one end of a mattress suture, passing through Poupart's ligament, is drawn. These ends are tied together firmly so approximating the ligament securely to the bone. A second suture is passed through the ligament higher up than the first and drawn through the same two holes. According to the width of the loop of this stitch the ligament can be more or less puckered and tightened over the ring. He closes the sac by bisecting it, interlocking the two halves and returning them through the ring where they are left to act as a buttress. Roux has also been carrying out closure of the ring by attaching Poupart's ligament directly to the bone; he employs a metal staple passing through the ligament and driven into the bone.

Among the rarer forms of hernia the properitoneal are seldom recognised during life. Many cases of reduction *en masse* may be due to the presence of a second properitoneal sac. The absence of definite swelling renders the diagnosis very difficult. E. H. Howlett[4] records an interesting case; the patient had complained of a "lump" which used to press forward midway between the anterior superior iliac spine on the right side and the middle line; for this he had worn a truss for years. He came under treatment for intestinal obstruction of five days' duration. A hernial sac was found passing upwards and outwards behind the transversalis fascia, this contained gangrenous gut which was resected. The symptoms did not subside and at the necropsy a second large sac, situated between the peritoneum and transversalis fascia and passing downwards to the pelvis and side of the bladder, was discovered; in this deeper sac lay the resected gut and ligated omentum. The neck of the superficial sac, opened at the operation, was situated in the outer wall of this deeper sac, so that the contents of the first sac were reduced into the second sac instead of into the abdominal cavity. Moschowitz[5] narrates a case of strangulated interstitial hernia of the inguino-superficial variety. The patient had an undescended testis, for which he wore a truss. The testis had apparently escaped from the external ring, and finding the entrance to the scrotum barred, had turned up on to the aponeurosis of the external oblique; the patient found he was more comfortable when the testis lay here; he consequently utilised his truss to keep the testis above the pad and lying under the integuments on the abdominal wall. He came under treatment with symptoms of strangulation. Torsion of the cord was diagnosed. At the operation a strangulated hernia into the funicular process was discovered, the neck of the sac and cord were sharply bent up over the margin of the external ring. On bringing down the testis and sac the contents slipped back. The testicle was satisfactorily placed in the scrotum and retained its position there.

A case of large retroperitoneal hernia into the fossa duodeno-jejunalis is described by Chas. T. Andrews,[6] he found it in an old male subject in the dissecting room. It contained nearly all the small intestine; apparently no symptoms were produced during life.

1 Dub. Jour. Med. Sci., Dec. 1902. 2 Med. Rec., Feb. 14, 1903.
3 Brit. Med. Jour., Nov. 8, 1902. 4 Quart. Med. Jour., Aug. 1902.
5 Med. Rec. Jan. 1903. 6 Lancet, Jan. 1903.

